# Evaluation of the Palutop+4 malaria rapid diagnostic test in a non-endemic setting

**DOI:** 10.1186/1475-2875-8-293

**Published:** 2009-12-12

**Authors:** David PJ van Dijk, Philippe Gillet, Erika Vlieghe, Lieselotte Cnops, Marjan van Esbroeck, Jan Jacobs

**Affiliations:** 1 Faculty of Health, Medicine and Life Sciences (FHML), Maastricht, The Netherlands; 2 Department of Clinical Sciences, Institute of Tropical Medicine (ITM), Unit of Tropical Laboratory Medicine, Nationalestraat 155, B 2000 Antwerpen, Belgium

## Abstract

**Background:**

Palutop+4 (All. Diag, Strasbourg, France), a four-band malaria rapid diagnostic test (malaria RDT) targeting the histidine-rich protein 2 (HRP-2), *Plasmodium vivax-*specific parasite lactate dehydrogenase (Pv-pLDH) and pan *Plasmodium*-specific pLDH (pan-pLDH) was evaluated in a non-endemic setting on stored whole blood samples from international travellers suspected of malaria.

**Methods:**

Microscopy corrected by PCR was the reference method. Samples include those infected by *Plasmodium falciparum *(n = 323), *Plasmodium vivax *(n = 97), *Plasmodium ovale *(n = 73) and *Plasmodium malariae *(n = 25) and 95 malaria negative samples.

**Results:**

The sensitivities for the diagnosis of *P. falciparum*, *P. vivax, P. malariae *and *P. ovale *were 85.1%, 66.0%, 32.0% and 5.5%. Sensitivities increased at higher parasite densities and reached 90.0% for *P. falciparum *>100/μl and 83.8% for *P. vivax *> 500/μl. Fourteen *P. falciparum *samples reacted with the Pv-pLDH line, one *P. vivax *sample with the HRP-2 line, and respectively two and four *P. ovale *and *P. malariae *samples reacted with the HRP-2 line. Two negative samples gave a signal with the HRP-2 line. Faint and weak line intensities were observed for 129/289 (44.6%) HRP-2 lines in *P. falciparum *samples, for 50/64 (78.1%) Pv-pLDH lines in *P. vivax *samples and for 9/13 (69.2%) pan-pLDH lines in *P. ovale *and *P. malariae *samples combined. Inter-observer reliabilities for positive and negative readings were excellent for the HRP-2 and Pv-pLDH lines (overall agreement > 92.0% and kappa-values for each pair of readers ≥ 0.88), and good for the pan-pLDH line (85.5% overall agreement and kappa-values ≥ 0.74).

**Conclusions:**

Palutop+4 performed moderately for the detection of *P. falciparum *and *P. vivax*, but sensitivities were lower than those of three-band malaria RDTs.

## Background

Malaria is a widespread and life-threatening disease. Each year, 10,000 malaria cases are reported among returned international travellers, and the real number of cases is estimated at 30,000 [1]. Prompt diagnosis is essential for the treatment and outcome, and malaria rapid diagnostic tests (malaria RDTs) may be of help to non-experienced laboratory staff in non-endemic settings [2,3].

Malaria RDTs are immunochromatographic tests targeting specific antigens of one or more *Plasmodium *species. Malaria RDTs are available as strips or cassettes, and display visible cherry-red to purple coloured control and test lines. The initially developed two-band malaria RDTs had, besides a control line, a *Plasmodium falciparum*-specific line targeting histidine-rich protein-2 (HRP-2) or *P. falciparum*-specific parasite lactate dehydrogenase (Pf-pLDH). Later developed three-band malaria RDTs additionally detected an antigen common to the four main *Plasmodium *species, such as aldolase or the pan *Plasmodium*-specific pLDH (pan-pLDH). In addition, a few four band malaria RDTs are on the market, such as Palutop+4 (All. Diag, Strasbourg, France). This malaria RDT detects three antigens: *P. falciparum*-specific HRP-2, *Plasmodium vivax*-specific pLDH (Pv-pLDH) and pan-pLDH. Four-band malaria RDTs may distinguish between infections with *P. falciparum*, *P. vivax *or another *Plasmodium *species and, therefore, the intended field of application includes non-endemic settings such as travel clinics. In this study, Palutop+4 was evaluated on stored blood samples of returned international travellers.

## Methods

### Study design

In this retrospective study, Palutop+4 was evaluated with a collection of stored samples obtained from international travellers. Tests were carried out in the reference laboratory of the Institute of Tropical Medicine (ITM) in Antwerp, Belgium. The study design was in compliance with the STARD guidelines for presentation of diagnostic studies [4].

### Patients and samples

Samples were selected from a collection of EDTA-blood samples stored at -70°C and obtained from patients presenting at the outpatient clinic of ITM. The patients were international travellers and, to a lesser extent, immigrants returning from visits to their native countries. In addition, samples sent by Belgian laboratories to ITM in the scope of the national reference function were included. The 530 samples collected at ITM, were aliquoted and frozen at -70°C the day of collection. Between collection and storage at -70°C, the samples remained for a maximum of 10 hours at ambient temperatures below 25°C. The 83 samples submitted by Belgian laboratories to ITM for second opinion and confirmation were sent by mail and had been exposed to ambient temperature for the period of shipment, which was generally less than 24 hours and ranged to a maximum of 48 hours. The delays of shipment and processing before storage at -70°C had been validated before and were compliant with routine laboratory procedures. In addition, samples from symptomatic patients without malaria parasites (as tested with microscopy and polymerase chain reaction (PCR)) were included.

### Reference method

Microscopy, corrected by PCR, was used as the reference method. Standard microscopy including determination of parasite density was performed [5]. Malaria diagnosis at Central Laboratory of Clinical Biology is accredited in accordance with the requirements of the ISO 15189:2007 norm. The laboratory technicians have received a detailed training and their performance and agreement are monitored by participation to internal and external quality control assessments. As a standard procedure, all slides with *Plasmodium *spp. are confirmed by a second blinded microscopist, as well as those slides that show results different from those obtained by RDTs and PCR. PCR analysis was performed on all discordant samples, with a species-specific real-time PCR as described previously [5].

### Test platform

Palutop+4 is a lateral flow immunochromatographic malaria RDT in a cassette format. Four lines are present, a control line which indicates whether the test is valid, a HRP-2 line, a Pv-pLDH line and a pan-pLDH line. According to the manufacturer's instructions, Palutop+4 can detect *P. falciparum*, *P. vivax, Plasmodium ovale *and *Plasmodium malariae*. Further it can distinguish between *P. falciparum *(unique HRP-2 line visible), *P. vivax *(unique Pv-pLDH line visible), the other two *Plasmodium *species (unique pan-pLDH line visible) or mixed *Plasmodium *infections (all other combinations of test lines). For the evaluation, test kits of two different lot numbers were used, 91055 and 91057. Test kits were stored at a dry place between 18°C and 30°C.

### Test procedure

Tests were performed according to the instructions of the manufacturer except that samples (5 μl) where loaded with a transfer pipette (Finnpipette, Helsinki, Finland) instead of the plastic loop supplied. In case the control line did not appear, the result was interpreted as invalid and the test was repeated. Test lines were scored for intensity as negative (no line visible), faint (barely visible line), weak (paler than the control line), medium (equal to the control line) or strong (stronger than the control line) [5]. Readings were performed 15 minutes after application of the sample and diluent, by three blinded observers. The results of the readings were based on consensus agreement, which means that a positive result was defined as a result read positive by at least two out of three different observers. Inter-reader reliabilities were assessed for positive and negative readings as well as for the intensity readings. To assess reproducibility, a panel of 16 samples (including four *P. falciparum*, four *P. vivax*, four *P. ovale *and four *P. malariae *samples) was tested on five successive occasions.

### Statistical analysis

Samples infected with *P. falciparum*, *P. vivax *and the two other *Plasmodium *species were considered separately. For *P. falciparum*, samples with pure gametocytaemia were included among the positive samples. Tables 1, 2 and 3 list the definitions used for calculation of test characteristics for *P. falciparum, P. vivax *and the two other *Plasmodium *species respectively. Sensitivity and specificity were calculated with 95% confidence intervals (CI) and differences were tested for significance using the Yates chi-square test or, when this was not possible, the Fisher exact probability test. A p-value of < 0.05 was considered significant. Reliabilities for positive and negative readings and line intensities were calculated as percentage agreements for all three readers and kappa values for each pair of readers. Associations between line intensity readings and parasite densities were assessed for strength of association with Cramer's *V *for categorical variables.

**Table 1:** Interpretation of test results for Palutop+4 for the detection of P. falciparum*

Test Line(s) visible	Species	
	***P. falciparum***	***P. vivax, P. ovale, P. malariae *or no parasites detected**
		
Only HRP-2 or HRP-2 + pan-pLDH	True positive	False positive or species-mismatch†
		
None or HRP-2 + Pv-pLDH or HRP-2 + Pv-pLDH + pan-pLDH or Only Pv-pLDH or Pv-pLDH + pan-pLDH or Only pan-pLDH	False negative or species-mismatch‡	True negative

†Non-*falciparum *species diagnosed as *P. falciparum*

‡*P. falciparum *diagnosed as non-*falciparum *species or a mixed *Plasmodium *spp. infection

**Table 2:** Interpretation of test results for Palutop+4 for the detection of *P. vivax*

	**Species**	
	
**Test Line(s) visible**	***P. vivax***	***P. falciparum, P. ovale, P. malariae *or no parasites detected**
		
Only Pv-pLDH or Pv-PLDH + pan-pLDH	True positive	False positive or species-mismatch†
		
None or Only HRP-2 or HRP-2 + Pv-pLDH or HRP-2 + pan-pLDH or HRP-2 + Pv-pLDH + pan-pLDH or Only pan-pLDH	False negative or species-mismatch‡	True negative

†Non-*vivax *species diagnosed as *P. vivax*

‡*P. vivax *diagnosed as non-*vivax *species or a mixed *Plasmodium *spp. Infection

**Table 3:** Interpretation of test results of Palutop+4 for the detection of *P. ovale *and *P. malariae*

**Test Line(s) visible**	**Species**	
	
	***P. ovale or P. malariae***	***P. falciparum, P. vivax *or no parasites detected**
		
Only pan-pLDH	True positive	False positive or species-mismatch†
		
Any other possibility except "only pan-pLDH"	False negative or species-mismatch‡	True negative

†*P. falciparum *or *P. vivax *diagnosed as *P. ovale or P. malariae*

‡*P. ovale *or *P. malariae *diagnosed as *P. falciparum *or *P. vivax *or a mixed infection with one of these *Plasmodium *spp.

### Duration of storage

To examine the effect of the duration of storage on the test performance, the sensitivity of samples obtained between 1995 and 2000 was compared with those obtained between 2001 and 2008.

### Ease of use

Three experienced laboratory technicians scored the ease of use of Palutop+4 test and the clarity of manufacturer's instructions with a standardized list [5].

### Ethical review

The study was reviewed and approved by the Institutional Review Board of ITM and by the Ethical Committee of Antwerp University, Belgium.

## Results

### Sample collection

A total of 613 samples was selected,. They had been collected from December 1995 to August 2008. According to microscopy and after correction by PCR analysis, 323 of these samples were positive for *P. falciparum*, 97 for *P. vivax*, 73 for *P. ovale *and 25 for *P. malariae*. In addition, 95 microscopic and PCR negative samples were included. The majority (270/323) of *P. falciparum *infections was acquired in Africa. They were obtained from 613 patients, with a male-to-female ratio of 2.07:1, and median age of 36.5 years (range 1 - 84 years). Only a minority (nine patients, 1.5%) were children less than five years old.

### Invalid test results

One of the 613 samples gave an invalid result at initial testing. Upon repetition, the test performed well.

### Sensitivity, specificity and species mismatch

RDTs were performed between November and December 2008. Table 4 lists the results of the three test lines and Table 5 lists the sensitivities for the different species and parasite densities. For the detection of *P. falciparum*, the overall sensitivity was 85.1% and was related to parasite densities, with values at parasite densities > 100/μl which were significantly higher as compared to those ≤ 100/μl (90.0% versus 67.9% respectively, p < 0.001). For the detection of *P. vivax*, the sensitivity was 66.0%, with higher values above and lower values below the parasite density breakpoint of 500/μl (83.8% versus 24.1%, p < 0.001). The sensitivities for the detection of *P. malariae *and *P. ovale *were 32.0% and 5.5% respectively. The differences between parasite densities below and above 500/μl did not reach statistical significance. The specificities for *P. falciparum*, *P. vivax *and *P. ovale/P. malariae *were 96.9% (94.0%-98.5%), 100% (99.1%-100%) and 100% (99.1%-100%) respectively. The two malaria negative patients with a false positive test result (Table 4) did not have a recent history of malaria. Species mismatch was observed in 21/518 *Plasmodium*-positive samples (4.1%, Table 4): fourteen *P. falciparum *samples reacted with all three test lines, one *P. vivax *sample reacted exclusively with the HRP-2 line, and respectively two and four *P. ovale *and *P. malariae *samples reacted with the HRP-2 line. In the case of *P. falciparum*, cross-reactions with the Pv-pLDH line occurred more frequently at parasite densities of ≥ 100,000/μl compared to lower parasite densities (9/61 versus 5/262, p < 0.001). There was no relation between the duration of storage of the samples and the sensitivity, specificity and line intensities of the test.

**Table 4:** Test results of all samples (n = 613)

**Samples**	**HRP-2 line positive**				**HRP-2 line negative**			
		
	**Pv-LDH line positive**		**Pv-pLDH line negative**		**Pv-LDH line positive**		**Pv-pLDH line negative**	
				
	**pan-pLDH line positive**	**pan-pLDH line negative**	**pan-pLDH line positive**	**pan-pLDH line negative**	**pan-pLDH line positive**	**pan-pLDH line negative**	**pan-pLDH line positive**	**pan-pLDH line negative**
								
***P. falciparum*** **(n = 323)**	14†	-	148	127	-	-	-	34
								
***P. vivax*** **(n = 97)**	-	-	-	1†	50	14	-	32
								
***P. ovale*** **(n = 73)**	-	-	-	2†	-	-	4	67
								
***P. malariae*** **(n = 25)**	-	-	1†	3†	-	-	8	13
								
**Negative** **(n = 95)**	-	-	-	2	-	-	-	93

†Special mismatch

**Table 5:** Sensitivity of Palutop+4 for the detection of all *Plasmodium *species related to parasite densities*

**Species**	**Numbers**	**Correctly identified by Palutop+4†**	**Sensitivity in % (95% CI)**
			
**All *P. falciparum *samples**	323	275	85.1 (80.7-88.7)
Pure gametocytaemia	17	12	70.6 (44.0-88.6)
Parasite density 0-100/μl	56	38	67.9 (53.9-79.4)
Parasite density 101-1000/μl	116	103	88.8 (81.3-93.7)
Parasite density >1000/μl	134	122	91.0 (84.6-95.1)
Parasite density >100/μl	250	225	90.0 (85.4-93.3)
			
**All *P. vivax *samples**	97	64	66.0 (55.6-75.1)
Parasite density ≤ 500/μl	29	7	24.1 (11.0-43.9)
Parasite density >500/μl	68	57	83.8 (72.4-91.3)
			
**All *P. ovale *samples**	73	4	5.5 (1.8-14.2)
Parasite density ≤ 500/μl	36	0	0 (0-12.0)
Parasite density >500/μl	37	4	10.8 (3.5-26.4)
			
**All *P. malariae *samples**	25	8	32.0 (15.7-53.6)
Parasite density ≤ 500/μl	9	2	22.2 (3.9-59.8)
Parasite density >500/μl	16	6	37.5 (16.3-64.1)

†A *P. falciparum *sample generating a unique HRP-2 line or both HRP-2 and pan-pLDH lines or a *P. vivax *sample generating a unique Pv-pLDH line or both Pv-pLDH and pan-pLDH lines or a *P. ovale *or *P. malariae *sample generating a unique pan-pLDH line

### Line intensities

For *P. falciparum *samples, 129/289 (44.6%) HRP-2 readings gave faint or weak line intensities. For *P. vivax *samples, faint and weak line intensities accounted for 78.1% (50/64) positive Pv-pLDH line readings, as well as for all (n = 50) positive pan-pLDH line readings. For the *P. ovale *and *P. malariae *samples combined, two and seven out of 13 positive pan-pLDH readings were faint and weak respectively, the remaining four showed medium line intensities. Line intensities for the HRP-2 and pan-pLDH lines were related to parasite densities (HRP-2: *V *= 0.568, p < 0.001; Pv-pLDH: *V *= 0.293, p = 0.14; pan-pLDH: *V *= 0.397, p < 0.001).

Of interest is that in the case of positive *P. falciparum *samples, the unique presence of a HRP-2 line pointed in most cases (90.5%, 115/127 samples) to a parasite density below 1000/μl. The co-presence of both HRP-2 and pan-pLDH lines predicted parasite densities above 1000/μl in 115/148 (77.7%) of samples.

### Inter-reader reliability and reproducibility

For the HRP-2 line and the Pv-pLDH line, the inter-reader reliability for positive and negative test results was excellent: for the HRP-2 line, there was 92.0% agreement between the three readers and kappa values between the three pairs of readers were 0.89, 0.88 and 0.88. For the Pv-pLDH line, overall agreement was 96.4%, and kappa values were 0.91, 0.88 and 0.90. The inter-reader reliability for the pan-pLDH line was lower, with 85.5% overall agreement and kappa values of 0.74, 0.76 and 0.88 for the three pairs of readers.

For the line intensity results, the overall agreement for the HRP-2 line was 69.0% with kappa values of 0.67, 0.72, and 0.67 for the three pairs of readers. For the Pv-pLDH line intensity, the overall agreement between the three readers was 91.4% with kappa values of 0.81, 0.72 and 0.73 for the three pairs of readers. For the pan-pLDH line intensity, the overall agreement between the three readers was 71.1% with kappa values of 0.63, 0.66 and 0.70 for the three pairs of readers.

Test results (positive or negative) and line intensity readings were reproducible. Consistent test results upon five test occasions were obtained for 12 out of 16 samples. From the four remaining samples, three and one had consistent results upon four and three occasions respectively. These four samples included three *P. malariae *samples that showed positive and negative pan-pLDH lines upon successive testing and a single *P. falciparum *sample that showed an additional Pv-pLDH line on a single test occasion. All inconsistent results for line intensity readings had discordances only within one category of difference in line intensity.

### Duration of storage

There was no significant difference between test sensitivities of samples that had been stored for long versus shorter periods, for any of the four *Plasmodium *species (Table 6).

**Table 6:** Comparison of test sensitivities between samples that had been stored for long and short periods

**Species**	**Year**	**Numbers**	**Correctly identified by Palutop**	**Sensitivity in %** **(95% CI)**	**p =**
					
*P. falciparum*	1995-2000	109	95	87,2 (79,1-92,5)	
					
	2001-2008	214	180	84,1 (78,4-88,6)	0,467
					
*P. vivax*	1995-2000	46	32	69,6 (54,1-81,8)	
					
	2001-2008	51	32	62,8 (48,1-75,5)	0,479
					
*P. ovale*	1995-2000	32	2	6,3 (1,1-22,2)	
					
	2001-2008	41	2	4,9 (0,8-17,8)	0,593
					
*P. malariae*	1995-2000	7	2	28,6 (5.1-69,7)	
					
	2001-2008	18	6	33,3 (14,4-58,8)	0,607

### Ease of use

Palutop+4 was scored as easy to use and practical, and the instructions were scored as clear and simple to perform by all three technicians. However, in roughly one third of samples, the clearance of the test strip was not optimal and the strip remained red-coloured after completion of the test procedure.

## Discussion

This retrospective study showed that Palutop+4 performed moderately well for the detection of *P. falciparum *and *P. vivax *and poorly for the detection of *P. ovale *and *P. malariae*. Sensitivities for *P. falciparum *and *P. vivax *at the parasite density breakpoints of 100/μl and 500/μl were 90.0% and 83.8%, respectively. Species mismatches occurred in 4.1% of *Plasmodium*-positive samples. Test results were reliable and reproducible and the test was scored as practical.

For this study, several limitations should be considered. For instance, its retrospective design impeded the possibility to test patients for known causes of false positive test results such as the presence of the rheumatoid factor [6]. Further, a transfer pipette was used instead of the test's application loop, thereby biasing any test errors due to volume error [7]. Likewise, tests were performed in a reference setting and results should not be extrapolated to field settings [8]. Furthermore, the storage-time of the samples could affect antigen stability [7], although in the present study the samples were not exposed to repeated thawing and freezing procedures. There were no significant differences in sensitivities between samples that had been stored for long versus short periods for any of the four *Plasmodium *species. Other evaluations, including the World Health Organization (WHO) Malaria RDT Evaluation Programme, also used stored samples [9]. In addition, for HRP-2, a prospective study revealed similar results between fresh and stored samples for HRP-2 detection: 125 samples were tested upon collection and after a storage duration of one to three years they were tested again. In only six of the 125 samples the results were discordant [10]. Finally, it should be noted that the present study used stringent criteria for defining test characteristics: as an example, the 14 *P. falciparum *samples that showed both the HRP-2 line and the Pv-pLDH line were scored as "species mismatches", although the error of diagnosing a mixed *P. falciparum *- *P. vivax *infection instead of a pure *P. falciparum *infection can be considered as a minor error. If these 14 results would have been scored as correct, sensitivity would increase to 89.5%. Although most papers on malaria RDTs do not describe in detail the criteria for defining test characteristics, it can be assumed that a previous study on Palutop+4 [11] scored this minor species-mismatch as correct, thereby explaining for the higher sensitivity.

The WHO list of malaria RDT manufacturers with adequate evidence of good manufacturing practice published online [12] actually includes seven four-band malaria RDTs. Palutop+4 is the only test for which published evaluations are available. In an endemic field setting, Rakotonirina *et al *found sensitivities for Palutop+4 of 95.4% and 94.2% for *P. falciparum *and *P. vivax*, respectively [11]. Unlike the present study, they did not include *P. ovale *or *P. malariae *positive patients. The present study showed sensitivities for *P. falciparum *and *P. vivax *which are lower as compared to their findings: apart from the possibility of a different definition of test characteristics discussed above, these lower results may also be due to a higher number of samples with low parasite densities and pure gametocytaemia. Further the present study included a higher amount of samples (323 versus 87 *P. falciparum *samples and 97 versus 17 *P. vivax *samples), thereby generating smaller confidence intervals. As in the present study, Rakotonirina *et al *also demonstrated species mismatch for one out of 87 *P. falciparum *samples (diagnosed as *P. ovale *or *P. malariae*) and two out of 17 *P. vivax *samples (one diagnosed as *P. falciparum *and one as *P. ovale *or *P. malariae*) [11].

Compared to other malaria RDTs in a non-endemic setting, Palutop+4 has a lower sensitivity for the detection of *P. falciparum*: reported sensitivities for two- and three-band malaria RDTs ranged from 87.5%-99.0% [13-18], with one exception of 76.2% [19]. In addition, the present study found the sensitivity of Palutop+4 at parasite densities above 100/μl to be lower than the 95% value recommended by the WHO [20]. The sensitivity for the detection of *P. vivax *(66.0%) is in line with other malaria RDTs. For the pan-pLDH, sensitivities of 35.3%[20] and 62.0%-95.0% [13,15,17] and for the aldolase, sensitivities of 46.0%-93.0% [15] have been reported. Studies in endemic settings that evaluated a dedicated Pv-pLDH two-band malaria RDT reported overall sensitivities of 96.4% and 93.4% [21,22], and in a comparable study design in the Antwerp laboratory, this malaria RDT showed an overall sensitivity of 88.0% for the diagnosis of *P. vivax *[23]. For *P. ovale *and *P. malariae*, it is more difficult to make comparisons, as sensitivities vary a lot among different studies, with values for pan-pLDH and aldolase ranging from 36.0% to 95.0% and from 7.0% to 80.0% respectively [13,15]. Part of the lower sensitivity of Palutop+4 for these species may be explained by the four-band design: optimal conditions, such as the pH and ionic strength of the diluent and coating of the nitrocellulose strips, must be met for three different antigen-antibody combinations, with an inevitable trade-off in test characteristics for one or more of the target antigens. Regarding species mismatches, cross-reactions of *P. falciparum *with high parasite density and the Pv-pLDH line have been described earlier [23]. Also, the number of *P. malariae *cross-reactions with the HRP-2 line was similar to other findings [5]. Cross-reactions for *P. vivax *or *P. ovale *are rarely observed [24].

Although Palutop+4 proved to be reliable and reproducible, most of the test line intensities, in particular those for Pv-pLDH and pan-pLDH, scored faint or weak. In addition, there was incomplete clearance of the test strips in about one third of specimens. These two facts may interfere with correct reading: misinterpretation of faint line intensities as negative test results is a frequent pitfall for inexperienced staff both in endemic and non-endemic settings [17,25]. Although the unique presence of the HRP-2 line and its co-presence with the pan-pLDH line were indicative for the parasite density, this prediction was not as strict and conclusive as observed in other malaria RDTs [5,26].

## Conclusions

In view of its theoretical ability to diagnose and differentiate among at least three of the four common *Plasmodium *species, the intended use of Palutop+4 is in non-endemic settings. However, the lower sensitivities of Palutop+4 for the *P. ovale *and *P. malariae *species must be taken into account and compared to three-band malaria RDTs. The four-band design tends to have a lower sensitivity for the detection of *P. falciparum *and *P. vivax*, even at higher parasite densities. Further improvement of test conditions may favour the use of four-band malaria RDTs in the future.

## Abbreviations

Ag: Antigen; CI: Confidence interval; DNA: Desoxy-ribonucleic acid; EDTA: Ethylene damine tetra-acetic acid; FHML: Faculty of Health Medicine and Life Sciences, Maastricht; HRP-2: Histidine-rich protein 2; ITM: Institute of Tropical Medicine; RDT(s): Rapid diagnostic test(s); P.: *Plasmodium*; Pan-pLDH: pan *Plasmodium*-specific parasite lactate dehydrogenase; PCR: Polymerase chain reaction; Pf-pLDH: *Plasmodium falciparum-*specific parasite lactate dehydrogenase; Pv-pLDH: *Plasmodium vivax*-specific parasite lactate dehydrogenase; WHO: World Health Organization.

## Competing interests

The authors declare that they have no competing interests.

## Authors' contributions

DvD, PG and JJ designed the study protocol. MvE and EV organized prospective sample collection. DvD and PG carried out the test evaluations, LC performed PCR analysis. DvD, PG and JJ analyzed and interpreted the results and drafted the manuscript. DvD performed statistical analysis. All authors contributed to the discussion of the results and the redaction of the manuscript, they all approved the final manuscript.

## References

[B1] World Health Organization International travel and health, situation as on 1 January 2009 2009

[B2] Hanscheid T Current strategies to avoid misdiagnosis of malaria Clin Microbiol Infect 2003 9 497 504 10.1046/j.1469-0691.2003.00640.x 12848724

[B3] Moody A Rapid diagnostic tests for malaria parasites Clin Microbiol Rev 2002 15 66 78 10.1128/CMR.15.1.66-78.2002 11781267 PMC118060

[B4] Bossuyt PM Reitsma JB Bruns DE Gatsonis CA Glasziou PP Irwig LM Moher D Rennie D de Vet HC Lijmer JG The STARD statement for reporting studies of diagnostic accuracy: explanation and elaboration. The Standards for Reporting of Diagnostic Accuracy Group Croat Med J 2003 44 639 650 14515429

[B5] Palen M Van der Gillet P Bottieau E Cnops L Van Esbroeck M Jacobs J Test characteristics of two rapid antigen detection tests (SD FK50 and SD FK60) for the diagnosis of malaria in returned travellers Malar J 2009 8 90 10.1186/1475-2875-8-90 19416497 PMC2688521

[B6] Iqbal J Sher A Rab A *Plasmodium falciparum *histidine-rich protein 2-based immunocapture diagnostic assay for malaria: cross-reactivity with rheumatoid factors J Clin Microbiol 2000 38 1184 1186 10699018 10.1128/jcm.38.3.1184-1186.2000 PMC86370

[B7] Bell D Peeling RW Evaluation of rapid diagnostic tests: malaria Nat Rev Microbiol 2006 4 S34 S38 10.1038/nrmicro1524 17034070

[B8] Murray CK Gasser RA Jr Magill AJ Miller RS Update on rapid diagnostic testing for malaria Clin Microbiol Rev 2008 21 97 110 10.1128/CMR.00035-07 18202438 PMC2223842

[B9] World Health Organization Malaria Rapid Diagnostic Test Performance; Results of WHO product testing of malaria RDTs: Round 1 (2008) 2009 http://www.finddiagnostics.org/resource-centre/reports_brochures/malaria-diagnostics-report-2009.html

[B10] Mayxay M Pukrittayakamee S Chotivanich K Looareesuwan S White NJ Persistence of Plasmodium falciparum HRP-2 in successfully treated acute falciparum malaria Trans R Soc Trop Med Hyg 2001 95 179 182 10.1016/S0035-9203(01)90156-7 11355555

[B11] Rakotonirina H Barnadas C Raherijafy R Andrianantenaina H Ratsimbasoa A Randrianasolo L Jahevitra M Andriantsoanirina V Menard D Accuracy and reliability of malaria diagnostic techniques for guiding febrile outpatient treatment in malaria-endemic countries Am J Trop Med Hyg 2008 78 217 221 18256418

[B12] World Health Organization List of known commercially-available antigen-detecting malaria RDTs with adequate evidence of good manufacturing practice 2009 http://www.wpro.who.int/NR/rdonlyres/990245C0-F157-417A-90C7-B08A7E1A50BA/0/TotallistofISO131485criteria_Rev_24MAR09.pdf

[B13] De Monbrison F Gerome P Chaulet JF Wallon M Picot S Peyron F Comparative diagnostic performance of two commercial rapid tests for malaria in a non-endemic area Eur J Clin Microbiol Infect Dis 2004 23 784 786 10.1007/s10096-004-1202-9 15452770

[B14] Durand F Crassous B Fricker-Hidalgo H Carpentier F Brion JP Grillot R Pelloux H Performance of the Now Malaria rapid diagnostic test with returned travellers: a 2-year retrospective study in a French teaching hospital Clin Microbiol Infect 2005 11 903 907 10.1111/j.1469-0691.2005.01253.x 16216106

[B15] Marx A Pewsner D Egger M Nuesch R Bucher HC Genton B Hatz C Juni P Meta-analysis: accuracy of rapid tests for malaria in travelers returning from endemic areas Ann Intern Med 2005 142 836 846 15897534 10.7326/0003-4819-142-10-200505170-00009

[B16] Richardson DC Ciach M Zhong KJ Crandall I Kain KC Evaluation of the Makromed dipstick assay versus PCR for diagnosis of *Plasmodium falciparum *malaria in returned travelers J Clin Microbiol 2002 40 4528 4530 10.1128/JCM.40.12.4528-4530.2002 12454146 PMC154650

[B17] Wiese L Bruun B Baek L Friis-Moller A Gahrn-Hansen B Hansen J Heltberg O Hojbjerg T Hornstrup MK Kvinesdal B Gomme G Kurtzhals JA Bedside diagnosis of imported malaria using the Binax Now malaria antigen detection test Scand J Infect Dis 2006 38 1063 1068 10.1080/00365540600818011 17148078

[B18] Wongsrichanalai C Barcus MJ Muth S Sutamihardja A Wernsdorfer WH A review of malaria diagnostic tools: microscopy and rapid diagnostic test (RDT) Am J Trop Med Hyg 2007 77 119 127 18165483

[B19] Grobusch MP Hanscheid T Gobels K Slevogt H Zoller T Rogler G Teichmann D Comparison of three antigen detection tests for diagnosis and follow-up of falciparum malaria in travellers returning to Berlin, Germany Parasitol Res 2003 89 354 357 12632146 10.1007/s00436-002-0764-7

[B20] World Health Organization Regional Office for theWestern Pacific 2003 Malaria Rapid Diagnosis: Making it Work. Meeting report 20-23 January 2003. Manila, the Philippines 2003 http://www.searo.who.int/LinkFiles/Malaria_MalariaRDT.pdf

[B21] Kim SH Nam MH Roh KH Park HC Nam DH Park GH Han ET Klein TA Lim CS Evaluation of a rapid diagnostic test specific for *Plasmodium vivax* Trop Med Int Health 2008 13 1495 1500 10.1111/j.1365-3156.2008.02163.x 18983278

[B22] Lee SW Jeon K Jeon BR Park I Rapid diagnosis of vivax malaria by the SD Bioline Malaria Antigen test when thrombocytopenia is present J Clin Microbiol 2008 46 939 942 10.1128/JCM.02110-07 18160449 PMC2268337

[B23] Gillet P Bosselaers K Cnops L Bottieau E Van Esbroeck M Jacobs J Evaluation of the SD FK70 malaria Ag *Plasmodium vivax *rapid diagnostic test in a non-endemic setting Malar J 2009 8 129 10.1186/1475-2875-8-129 19519915 PMC2700805

[B24] Tjitra E Suprianto S Dyer M Currie BJ Anstey NM Field evaluation of the ICT malaria P.f/P.v immunochromatographic test for detection of *Plasmodium falciparum *and *Plasmodium vivax *in patients with a presumptive clinical diagnosis of malaria in eastern Indonesia J Clin Microbiol 1999 37 2412 2417 10405377 10.1128/jcm.37.8.2412-2417.1999 PMC85241

[B25] Rennie W Phetsouvanh R Lupisan S Vanisaveth V Hongvanthong B Phompida S Alday P Fulache M Lumagui R Jorgensen P Bell D Harvey S Minimising human error in malaria rapid diagnosis: clarity of written instructions and health worker performance Trans R Soc Trop Med Hyg 2007 101 9 18 10.1016/j.trstmh.2006.03.011 17049572

[B26] Richter J Gobels K Muller-Stover I Hoppenheit B Haussinger D Co-reactivity of plasmodial histidine-rich protein 2 and aldolase on a combined immuno-chromographic-malaria dipstick (ICT) as a potential semi-quantitative marker of high *Plasmodium falciparum *parasitaemia Parasitol Res 2004 94 384 385 10.1007/s00436-004-1213-6 15549388

